# Resolutions of Hahnemannian Association of Pennsylvania

**Published:** 1888-09

**Authors:** Wm. Jefferson Guernsey

**Affiliations:** Secretary


### RESOLUTIONS OF THE HAHNEMANNIAN ASSOCIATION OF PENNSYLVANIA.

A special meeting of the "*Hahnemannian Association of Pennsylvania*" was held in the Continental Hotel, Philadelphia, July 21st, to take action on the death of Dr. Adolphus Fellger.

The President, Dr. C. Carleton Smith, was in the chair, and opened the meeting with the following eulogistic address:

"Gentlemen, the sad occurrence which brings us together on this occasion forcibly reminds us that death is again busy within our ranks. The announcement of the demise of our friend and colleague, Dr. Adolphus Fellger, did not come to us as a surprise. And yet in this instance, as in all instances of a similar kind, death brings sorrow to the heart and casts about us its deep, overhanging shadow.

"The man we mourn to-day was one of the few staunch defenders of the homœopathic faith. One of the few who was true and faithful to the trust imposed upon him, even unto the end. Loyal to his convictions, which were deep and abiding, loving the truth for truth's sake, he passed from our midst, dropping dead at the post of duty and at the front of the battle, leaving behind him a name and a memory which will never die."

The following preamble and resolutions were presented by the Committee appointed for that purpose, consisting of Dr. C.

Carleton Smith, President; Dr. John V. Allen, Dr. Walter M. James, Dr. E. J. Lee, Dr. Wm. Jefferson Guernsey, Dr. Mahlon Preston:

WHEREAS, This Association has learned, with sincere regret, that their late colleague, Dr. Adolphus Fellger, has been called to his rest. Therefore be it

*Resolved*, That this Association has lost an esteemed co-laborer, an able counselor, and a valued friend; the profession has been deprived of one of the most learned of its number, and the public at large of a skillful and untiring servant.

*Resolved*, That a copy of these resolutions be transmitted to his family, and that they be published in the *Public Ledger* of this city, and in the medical journals.

After a general expression of regret on the part of all present, that so able and trusty a follower of Hahnemann should have been taken, the meeting adjourned.

WM. JEFFERSON GUERNSEY,
*Secretary.*

